# The Relationship of Religiosity and Marital Satisfaction: The Role of Religious Commitment and Practices on Marital Satisfaction Among Pakistani Respondents

**DOI:** 10.3390/bs9030030

**Published:** 2019-03-20

**Authors:** Jaffar Aman, Jaffar Abbas, Mohammad Nurunnabi, Shaher Bano

**Affiliations:** 1 School of Sociology and Political Science, Shanghai University, Shanghai 200444, China; sbano@t.shu.edu.cn; 2 Antai College of Economics and Management (ACEM) / School of Media and Communication (SMC), Shanghai Jiao Tong University (SJTU), No. 800 Dongchuan Road, Minhang District, Shanghai 200240, China; 3 Department of Accounting, Prince Sultan University, Riyadh 11586, Saudi Arabia; 4 St Antony’s College, University of Oxford, Oxford OX2 6JF, UK

**Keywords:** spirituality, sociology of religion, marital satisfaction, religious practice and commitment

## Abstract

The sociology of religion focuses on an individual’s social and married life. This research performed the first focalized examination of the influence of spirituality and religiosity on the marital satisfaction of Pakistani Muslim couples and how religious commitment and religious practice strengthens the relationship of married couples. This study incorporates the Kansas Marital Satisfaction scale (KMSS), the Religious Commitment Inventory (RCI-10) and the Religious Practice scale to measure marital satisfaction. Survey questionnaires, including a survey invitation letter and an informed consent form, were sent to married couples residing in five urban areas of Pakistan. The sample consisted of 508 valid responses, 254 males and 254 females, exploring the respondent’s perception of their marital satisfaction. The data received were screened and tested through SPSS version 25. The first step of the data analysis was to examine the impact of religiosity variables (religious commitment, religious practice) on marital satisfaction. Findings indicated that religious commitment and religious practice are vital for a happy married life. The findings help explain the social dynamics of marital satisfaction in Pakistani culture. The results also indicated that religious commitment and religious practice strengthened and promoted marital satisfaction. This study is novel in the context of Pakistani culture and conclusions cannot be generalized to the whole population. Other religious factors may provide further research directions. The results of this study may help practitioners and decision-makers focusing on marital satisfaction issues.

## 1. Introduction

Religion and human life have a closer relationship in several societies around the world. Numerous scholars have described the indispensable role of religiosity practices and their impact on marital satisfaction. Researchers have examined the influence of religiosity in strengthening marital satisfaction; however, there is a shortage of studies seeking to understand the influence of religiosity on marital satisfaction in the Pakistani context [1]. In several societies, marriage is regarded as a religious sacrament as married couples typically pledge to spend their lives together in the eyes of God and marriage has been intimately associated with religion [2]. Religion emphasizes marriage and couples who believe in religion make a stronger marital commitment, which strengthens their marital relationship [3,4]. Previous studies have evidenced that religion plays an indispensable role in increasing marital satisfaction and has moderated the symptoms of the crises of human life [5,6]. Many previous studies focused on and investigated the idea that the relationship between religiosity level and marital satisfaction is positively associated; more religious married couples have a happier, more stable married life compared with other couples [3,4,7,8]. Most of the researchers to have examined the relationship between religiosity and marital satisfaction have come from the USA, Canada, and other first world countries; however, some studies have come from Turkey and Iran [9] and have revealed that religiosity is a vital predictor of marital satisfaction. In contrast, there is a gap in the literature in the context of the Pakistani Muslim community. This specific study claims to fill the critical research gap with an extensive investigation utilizing a quantitative research method.

Humans prefer to live in better societies and choose valuable life partners with which to create a happy and peaceful life. Marriage (wedlock or matrimony) is a recognized legal, social and ritual union with established obligations and rights. The standardized definition of the term marriage varies between cultures and regions but marriage is considered compulsory by many religions in order to pursue sexual activities. Hence, marriage is a contract between people that represents love in addition to the obligations of harmony, pleasure and the continuation of family relations. A study by Zahra Alghafli examined couples’ perceptions of Islamic teachings about marital satisfaction and family relations from an insider’s perspective and examined how participants reflected on and portrayed vital issues such as women’s fundamental rights, gender roles and the unity of family relationships. Marital commitment and satisfaction are important in improving the partners’ level of satisfaction in the assessment of various aspects of their marital relations [10]. Generally, the quality of marital commitment indicates the level of happiness and satisfaction of the individuals in their marital relationships. According to Zaloudek (2014), in the hierarchy of gender and family settings, husbands are regarded as the head of family relationships. This is also in line with Islamic principles; husbands are responsible for the family’s duties and obligations [11]. The term religiosity is not easily defined. Various researchers have addressed this notion in a broad sense, associating religious involvement and orientation. Religiosity shows several factors, including experiential, ceremonial, ritual, ideological, consequential, rational, practical, belief or creedal, moral, and cultural factors. According to sociologists, religion is seen as the practice and ideas of people, and a sense of being in a righteous place. Individuals’ behaviors usually do not match their practice and beliefs because there is a vast diversity in the definition of whether one is religiously minded or secure in practice [12]. According to Dinh et al. (2017), marital satisfaction is defined as personal feelings of happiness or pleasure and the spouses’ happiness considering all current aspects of the marriage [13]. Other studies have lacked empirical research in the Pakistani context of religion and the family snd in terms of alternative, globally significant faiths, such as Islam [14,15]. Social scientists should examine the influence of family life in non-Western religious traditions, such as Buddhism, Confucianism, Hinduism, and Islam.

The literature investigating marital satisfaction indicates that factors such as interaction levels, traditional gender roles, conflict management, problem-solving style, and marital romance influence marital satisfaction [16]. One study suggested [17] that categorically emphasizing any single specific factor cannot guarantee complete marital satisfaction. Several research studies examined the relationship between religiosity factors and marital satisfaction in different parts of the world. However, such a review from a Pakistani Muslim community perspective is a new contribution. A few research studies investigating the relationship between marital satisfaction, religiosity, and attachment style have been conducted in the United States [18]. However, in Pakistan, no empirical studies have examined the relationship between marital satisfaction and religiosity. This study investigates how religiosity (religious commitment and religious practice) impact the marital satisfaction of Pakistani Muslim couples. This study was intended to extend the current literature in the area of marital satisfaction and its relationship to religiosity, and was designed to examine the influence of religiosity on marital satisfaction. The current research investigated the interrelationships among religious commitment, religious practice and the marital satisfaction of married Pakistani Muslim couples. Marital satisfaction was studied as a combination of attachment style and religiosity [19]. Some scholars from the USA examined the association between marital satisfaction, religiosity and attachment style of couples in the USA, while another study determined this association in the European context [20]. Thus, our review was designed to investigate the influence of religiosity on marital satisfaction in the Pakistani context. The following research questions were raised to explore the relationship between the variables, including religious beliefs and the religious commitment of Pakistani married couples, to establish marital satisfaction. This research has investigated the questions through data analysis by applying statistical methods.

**RQ1:** *Do religious commitment and marital satisfaction correlate for Muslim couples in Pakistan?*

**RQ2:** *Does a correlation exist between religious practice and the marital satisfaction of Pakistani Muslim couples?*

## 2. Literature Review

Several social science researchers have considered marital satisfaction by examining two fundamental concepts, marital stability and marital quality, of married couples. Marital determination or marital stability refers to the length of marriage of a couple and whether the marriage dissolved by divorce or separation, or resulted in desertion or annulment. A decline in marital satisfaction and a high divorce rate are common phenomena in Western countries and some Asian countries, including Pakistan. Historically, marriage has a close relationship with religion because marriages are usually performed in religious synagogues and by religious leaders. Researchers who have studied the religious factors that enable couples to increase marital satisfaction have found that religiosity is closely related to marital satisfaction in Western cultures [21,22]. In this work, the researchers explored the general literature on marital satisfaction, including essential religiosity factors related to increased marital satisfaction. The religiosity literature, including factors related to marital satisfaction, was examined and previous studies of religiosity factors are discussed, attachment styles are also explained. In Pakistan, the traditional marriage system was an essential predictor of marital satisfaction [23]. Relatives, kin, or matchmakers who knew both the husband and wife arranged their marriage. A study showed [24] that, in an arranged marriage, the main characters were not the bride and groom but rather their parents, called the masters of the wedding ceremony. Pakistani people considered marriage to be the union of two families rather than that of two individuals. Most young people in Pakistan, however, no longer want to follow the traditional marriage ideology and pursue marital happiness and romantic love, while their parents focus on commitment or loyalty [25]. However, parents still play an essential role in the engagement and marriage of their children.

This study posits that the main reason for the divergent outcomes is the system of dualities at the nexus of the relationship between religion and family stability. Scholars have predominantly examined an association between religiosity and family relationships, which has revealed different outcomes, partially because some researchers tend to explore the benefits while other scholars have focused on the ways religiosity might be toxic to families and individuals [26]. The social science literature indicates that religiosity or religiousness might be both a boon and a bane to married couples, families, individuals, various communities, societies, cultures, and nations. Scholars have investigated both sides of religion, the greater good as well as horrific harm, and have found that the answers lie in both the dominant and conflicting aspects of religion. In case of power, but not divergence, religiousness could be either helpful or harmful, but not the both. If the impacts of religiosity could be established, the minimal influence of religion would not warrant serious attention. Thus, theories simultaneously considering the reasons that religiosity could help or harm might provide a more stable and balanced perspective [1,26,27,28]. In the previous research of Mahoney, Pargament, Tarakeshwar, and Swank (2001), they claimed that divorce risk would be lesser when couples were highly religious minded, that this facilitates a long-lasting and well-functioning marriage. In some cases, as their findings documented, the parents’ higher religiousness had a better impact on the children’s adjustment [29]. In another study, Marks interviewed seventy-six highly religious minded Muslim, Jewish, Mormon, and Christian married couples to examine the religions’ three dimensions (religious practice, spiritual practice and, and faith community) and their impact on marriage. Their study revealed that religiosity and harmony of faith in God and practice and beliefs prolonged the marital functioning of religious couples [30].

In the previous study, David and Stafford (2013) applied a religion and spirituality-based relational model, using the association of people with God, religious couples’ joint communication and their forgiveness behaviors as the marital satisfaction predictors. The study showed that one’s spiritual relationship with God is the indirectly critical factor for quality of marriage and seems to manifest itself between married couples in religious communication, which was directly associated with marital quality [31]. According to Reference [32], there are three fundamental perspectives on marriage: marriage as a ceremony, marriage as a concordat and marriage as an agreement. The perception of marriage as a ceremony derives from the Islamic custom. The perspective of marriage as a treaty is the predominant view of Islamic philosophy. The interpretation of the agreement is that marriage is a two-sided indenture that is willingly shaped, sustained, and dissolved (Köstenberger, 2004). The opinion of marriage as a contract suggests that marriage is a blessed union between husband and wife [33]. Reference [34] defines marital satisfaction as “one’s global and overall evaluations or attitudes toward the partner and the relationship.” Onsy, E. and M.M. Amer [35] contend that marriage begins with a high level of marital satisfaction but that marital satisfaction gradually declines as couples adjust to new marital circumstances. Marital satisfaction also decreases when the couple has children [15]. Thus, couples become less satisfied because they need to negotiate their responsibilities in their daily life. Rearing a child produces many stresses for married couples. There are no consistent findings on mid- and late-term marital satisfaction. Reference [36] argues that marital satisfaction increases in the later years of a marriage but contend that married couples do not show higher marital satisfaction in later years. Based on an intensive investigation of previous literature, Table 1 provides a brief description of the data sources and research methods adopted by various researchers from different parts of the world.

Marital satisfaction relates to many factors, sociodemographic variables, psychological factors and parenting, trends, psychopathology and physical health, or some combination of these factors (Bradbury, Fincham, and Beach, 2000). In the 1990s, studies of marital satisfaction focused strongly on marital perception—earlier studies of partners’ interpretations of undesirable behaviors and their autonomic physiology before communication conducted in the 1990s. According to the results of these studies, maladaptive attributes were closely related to increased negative behaviors when couples discussed their marital problems. Ferreira, M., et al. [37] examined, as a factor of marital satisfaction, micro and macro contexts, which identify the behavioral interactions between spouses from the perspective of the broader social context of couples’ lives. Micro contexts consist of the backgrounds and characteristics of the children and parents, along with life stressors and transitions. The most critical factors impacting marital relationships is parenthood. Children affect the marriage relationship between spouses. According to research on marriage and children, when children are relatively young, marital stability tends to increase while marital quality decreases [38]. The spouses’ backgrounds and characteristics impact the marital relationship. According to Reference [39], children with divorced parents may have poorer communication skills because of their parents’ divorce. Problematic behaviors mediate the association between parents’ divorce and their children’s divorce. Studies show that individuals who experienced depression when they were adolescents tended to marry earlier and have a higher rate of dissatisfaction than individuals who suffered other diagnoses. Most studies on couples in the middle of significant life and transition stressors have shown that difficult times often unite twosomes, thereby increasing their marital satisfaction. In the current literature, only a few research studies have investigated the relationships between religious commitment, religious practice, and marital satisfaction. According to Reference [40], there is a lack of research on the relationships between marital satisfaction and religiosity. However, a small study on this subject was identified and it showed a connection between the three variables [41]. Previous sociological studies have revealed that valuing religiosity level and the regular practice of religion have an association with better marital stability, greater marital satisfaction and an increased inclination to get married [42]. In another study, Madanian et al. (2013) claimed that married couples who sought and acknowledged their divine purpose of marriage showed more likelihood to collaborate, to maintain a higher level of marital adjustment and, ultimately, tended to perceive more advantages from the wedding [43].

These authors analyzed assortative mating by examining the similarity between spouses with specific characteristics (p. 1029–1030). Their study included 291 married couples who took part in the Iowa Marital Assessment Project (IMAP). They used correlations to measure the data between variables and found that couples who were similarly religious showed little similarity in attachment. Although some couples had similar levels of religiousness or affection, the couples’ similarities had little influence on their marital satisfaction [44]. Currently, family and marital life research cover a variety of tactics, including the merging of hypothetical possibility or outlines when examining marital relationships. Based on the goal of this research, the researchers employed a commitment theory to address the research objectives. Commitment is a concept that is fundamental to understanding the maintenance of human connection. Several typologies and commitment theories have been presented by behavioral and social scientists over the last several decades. George Levinger, who introduced the theory of commitment, was primarily interested in understanding the processes involved in both keeping relationships (particularly marriages) together and breaking them apart. A previous study explored the patterns of cultural identity, the sociodemographic and family relationship context of young adult Arab Muslim-American [45]. One study examined the family field within South Asian Muslim communities in the UK. The study sought to understand how parents pass on their values to their children. Research suggested that parents mobilized Islamic teachings in the transmission of morality, support of child education and the reinforcement family relationships [46]. Abdel-Khalek (2009) conducted a study to explore the relationship between religiosity, self-esteem, subjective well-being (SWB), and anxiety among Muslim Kuwaiti adolescents [47]. Eid (2011) investigated the association of religiosity, self-ratings of happiness, mental health, physical health, satisfaction with life, and depression of Kuwaiti (N = 1937) and Palestinian (N = 1009) Muslim children and adolescents [48]. Another study identified other highly loaded factors and labeled them well-being, mental health, and religiosity. Stepwise regression revealed that the key predictors of religiosity included happiness, satisfaction, self-esteem, and mental health in different combinations [49]. Zahra Alghafli (2017) examined the Muslim practice of wearing the hijab, the covering of a woman’s head and body. The research examined the hijab as a religious commitment [50].

Despite the progressively multiethnic and cultural influence of contemporary religious and social life, very few research studies have compared the real experiences of conservative religious females across the faith traditions [51]. Early commitment theories have emphasized the positive factors that typically led individuals to continue their lives in a relationship. The degree of love and satisfaction are important factors in keeping couples together [52]. On the other hand, later theories, while continuing the critical characteristics, focused on the positive factors that prevented individuals from leaving the marital relationships, such as societal disapproval of divorce or fear of starting the relationship process over with a new life partner [53]. At present, the most prominent existing theories describing relationship commitment include the investment model of Caryl Rusbult, George Levinger’s cohesiveness theory, and tripartite typology by Michael Johnson [53]. Though these approaches are different, they share some common factors, such as the notion of continuing relationships because of the elements that draw people to stay with their partners, and these factors prevent individuals from ending an association [54]. Regardless of the support of a theory, commitment is taken as an essential variable by the researchers of relationship studies for various reasons. First, it is taken as an indispensable motivational variable because of the strong influence of motivation and cognition on the relationship [55]. Second, the commitment is shown by the enactment of a variety of essential relationship maintenance behaviors. Behaviors including forgiveness, accommodation, or willingness to sacrifice for partners help sustain and maintain ties and are the factors that keep partners together [56]. The strengths of this theory can incorporate several research variables that have consequences for marital satisfaction and can distinguish marital satisfaction from marital stability.

**Figure 1:**
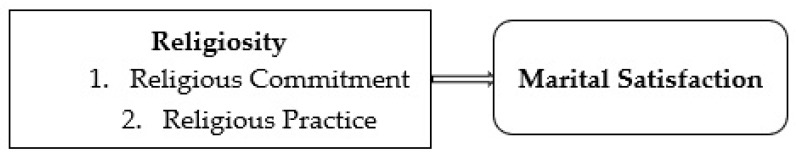
Conceptual Model.

Figure 1 Shows Pakistani couples religiosity, religious commitment & practice on marital satisfaction [50].

## 3. Research Hypothesis

**Hypothesis** **1.** *The first hypothesis in this study stated that Pakistani Muslim couples who had higher religious commitment would experience higher marital satisfaction.*

**Hypothesis** **2.** *The second hypothesis suggested that Pakistani Muslim couples who had firm religious practices would experience higher marital satisfaction.*

## 4. Research Methodology

In the current study, a detailed, point-by-point, literature review was conducted to develop a theoretical understanding of the theme, which included the utilization of data from journals and books. The present study examined the relationships between religiosity variables, such as religious commitment and religious practice, on the marital satisfaction of Pakistani Muslim couples living in selected urban areas of Pakistan. The primary purpose of this specific research was to investigate the factors of religiosity that influence the level of marital satisfaction of Pakistani Muslim couples. Quantitative analysis was used to assess attitudes, opinions, processes, and other measurable data from the many groups affected by the phenomena of interest. This study utilized an anonymous survey design to investigate the relationships between the variables. The respondents were over 20 years old and were married couples who had not experienced divorce or separation. The researcher examined the participants’ backgrounds, in terms of age, level of education, and number of years married. Participants volunteered to take this survey. Regarding factor analysis, the study identified two factors in the RCI-10. The first factor, comprising six items, measured intrapersonal religious commitment and the second factor, involving four elements, measured interpersonal religious commitment. The Kansas Marital Satisfaction scale (KMSS), a widely used instrument for assessing marital satisfaction, and the Religious Practice scale, adopted from Farahani and Musa and having four items, were used.

### 4.1. Research Procedures

This sample comprised 508 married couples based on both genders and researchers collected data with the help of the co-authors. Before administering the survey package, the researcher had an initial meeting with male and female respondents who showed a willingness to complete the survey. The purpose of this meeting was to explain the contents and procedure of the survey. The survey package included the following documents: an invitation letter for this survey, an informed consent information form, and demographic questionnaires to assess the marital satisfaction of Pakistani Muslim married couples. After the demographic questionnaires were administered, the sequence of the survey was rearranged in a different order. The researcher explained the entire poll process and how the participants could answer the demographic questionnaires. The researchers also demonstrated the instruments that would apply in this research study. Each respondent filled out survey forms in approximately 30 minutes. After completion of surveys, the researchers collected them from the participants and entered the data in SPSS software version 25, after the screening process.

### 4.2. Results of the Research

This unique study in Pakistani context aimed to examine the connections between religious commitment, religious practice, and the marital satisfaction of Pakistani Muslim couples. To accomplish this objective, the researchers analyzed the survey data using several statistical methods to obtain the analytical results of the samples and tested two research questions [57].

### 4.3. Descriptive Statistics

The demographic questionnaires used in this study covered the respondent’s age, gender and place of residence. The sample size of this study was 508 and comprised 254 males (50%) and 254 females (50%). The age factor was investigated according to the ranges: (1) 20–29, (2) 30–39, (3) 40–49, and (4) >50. The findings showed that 26.31% of respondents were aged 20 to 29 years, 46.05% were in the age bracket of 30-39 years, 21.38% were between 40 and 49, and only 5.59% were over 50 years of age. Regarding the collection of data from different fields, 23.0% of the respondents were from education sector, 22.06% were from the health sector, 13.08% were from the business sector, 15.07% were from households, and 26.78% were from public places. Statistics on the marriage duration of couples ranged from 1–4, 5–9, 10–14, 15–19 and 20–25 years. The biggest group, 39.30%, was for 5–9 years of marriage, and the smallest group, 4.30%, was for marriages lasting from 20–25 years. Regarding the region of the respondents, 16.44% were from Gilgit-Baltistan, 39.47% were from the Punjab province, 9.86% were from Baluchistan, 16.44% were from Khyber Pukhtonkha (KPK), and 17.79% were from the Sindh province.

Table 1 Displays the final results of all constructs, wherein the reliability is calculated. The Cronbach alpha values of marital commitment (MC = 0.797), marital satisfaction (MS = 0.750), and religious practice (RP = 0.712) indicate that the calculated values of the questionnaire show a satisfactory level of reliability.

### 4.4. Descriptive Statistics of Measurements

The statistical attributes of the predictor and criterion variables are presented in the tables below. The demographic attributes consisted of the number of participants, mean item scores (M), standard deviations (SD) and standardized Cronbach’s alpha (α). The questionnaires for marital satisfaction comprised four questions; religious practice included four and the variables of religious commitment were measured with a sub-scale of ten. All mean and standard deviation scores are shown below Table 2, Table 3 and Table 4, and the statistical results for each item is presented.

Table 5 reveals correlational results and findings were significant at the 0.01 level (2-tailed). Table 5 above illustrates the correlational relationships between the variables of the target population. Table 5 portrays these variables, discussed in depth, and has provided a detailed picture of the two-hypotheses raised in this study. In the discussion section, this study discussed the two main questions comprehensively.

Table 6 shows the simple regression analysis computed on the predictor variable of religiosity (religious commitment and practice) with the criterion variables of marital satisfaction. According to the results of correlation and simple regression, religious commitment and spiritual practice positively and significantly correlated with marital satisfaction.

## 5. Hypothesis Testing

The first hypothesis in this study stated that Pakistani Muslim couples who had higher religious commitment would experience higher marital satisfaction. The correlation analysis showed that religious commitment significantly correlated with marital happiness and values (r = 0.172**, *p* < 0.000). The results of this analysis revealed that marital satisfaction significantly associated with religious-commitment. Hence, the first hypothesis of this study was supported by the results.

The second hypothesis suggested that Pakistani Muslim couples who had firm religious practices would experience higher marital satisfaction. According to the findings of the correlation analysis, practice and beliefs significantly and positively correlated with the marital satisfaction of married couples (r = 0.271^**^, *p* < 0.000). Hence, the second hypothesis of this study was supported by the results.

## 6. Discussion and Conclusions

This study, aimed to investigate the role of religiousness in Pakistani society and the sociology of religion, was helpful in this context. Sociologists and anthropologists seek to describe the critical importance of the branch of religious sociology and how it concentrates on individuals’ lifestyles in society. The society of Pakistan has theological implications as 97% of its population is Muslim, and almost 2.5% is Christian. In Pakistani culture, people try to practice religion, and the findings of this study are helpful for policymakers and decision-making authorities. Henceforward, researchers should concentrate on individual’s practices and beliefs and their social behavior. Sociologists describe how social factors affect religion in the community and the sociology of religion emphasizes the social and marital lifestyles of people. The primary purpose of this unique study was to investigate the relationships between the identified variables of religiosity (religious commitment, religious practice) and marital satisfaction of married Muslim couples residing in five urban areas of Pakistan. This study examined the influence of religiosity and its components, religious commitment and religious practice, on marital satisfaction by applying correlational analyses. Religiosity was closely associated with the marital satisfaction of married individuals [58]. In Islamic society and Islamic terms, marriage is considered a religious sacrament in which couples swear devotion in front of God. Religiously committed couples appear to have better relationships with their spouses. Earlier studies, conducted in Western countries, considered marital satisfaction and the effects of religion on gender roles. For example, a survey by Zahra Alghafli examined perceptions of Islamic teachings and the family relationships of married couples from an insider’s perspective, and reflected on critical issues such as the fundamental rights of women, classifying gender roles and family relationships. An earlier qualitative study of Alghafli et al. (2014) examined Islam’s role on marital satisfaction and family associations from the insider’s perspective. They presented respondent’s reflections on sensitive issues such as gender roles, the rights of women, and marital stability and unity [10].

Most research investigating marital satisfaction has, in the past, focused on Caucasians and African Americans [59]. One study, conducted by Reference [60], asserted that since the late 1980s, researchers have begun to publish the results of studies of marital satisfaction of Japanese, Chinese, Indian, and Korean ethnic groups. Relatively few studies on religiosity, attachment style, and marital satisfaction have been conducted on Christian couples in the United States. This study demonstrated that religious Pakistani married couples tend to manage their stress, depression, anxiety, and physical illness better than non-religious couples do. Several previous studies on religion and marriage found that more religious couples had a happier and more stable marital life than other married couples in Islamic society in Pakistan [61]. In the context of Pakistan, few Pakistani researchers have examined the role of religion on the well-being of individuals or society in Pakistan. However, their studies have focused on a specific domain of religiosity and its impact on Pakistani society. No previous research has measured the relationships between religious commitment, religious practice, and marital satisfaction in the context of Pakistan. This study utilized a survey method to investigate the relationships between the predictor variables of religiosity with the criterion variable of marital satisfaction. This unique study, focusing on Pakistani married couples, examined the impact of religiosity, practice and beliefs, religious commitment and secure adult attachment style on the marital satisfaction of Pakistani Muslim couples. This study investigated the topic in the context of Pakistan, in which it is rare to fine literature on the religiosity, practice and beliefs and relationship, in terms of marital satisfaction concerning [29,30,31]. Various scholars have studied this question in a western context and in other parts of the world. The variables of this current study are novel because they concern Pakistani society. The results of this study showed that there was a significant trend in religiosity, and its components of religious commitment, and religious practice and the marital satisfaction of married Pakistani couples.

The findings of this study are in line with previous research, showing that more religious couples reported happier and more stable married lives than did other married couples [12]. According to the literature, religiosity creates marital satisfaction and intimacy among married couples and supports the importance of marriage, which creates marital commitment among spouses. Thus, religiosity leads to a satisfied and happy marriage. According to Austin, Macdonald, and MacLeod (2018), divorce has a negative impact. Religious couples have negative feelings about divorce and are willing to sacrifice for each other to maintain their marriage [62]. The study results demonstrated that religiosity and its components, religious commitment and religious practice along with influential factors, were associated with the marital satisfaction of married Pakistani couples. One possible explanation for the findings of this study can be found in a comparison with Watson and colleagues’ (2004) study, which examined, with a different design, the religious commitment of couples. The study by Watson and colleagues (2004) applied a correlation measurement to examine the association between relationships and attachment styles. They also examined the religiousness of married couples. However, this study also engaged a correlational method to investigate the relationships between religious commitment, religious practice, and the marital satisfaction of Pakistani married couples. The findings of this work are novel in the context of Pakistan and may contribute to various practice areas that, in future research, can adopt a broader scope, larger sample size, and different variables.

## 7. Limitations and Suggestions

Concerning the limitations of this study, its population included only married Pakistani Muslim couples from five urban areas of Pakistan. Therefore, future research may need more variability with different denominations to investigate the marital satisfaction of Pakistani Muslim couples. This sample size cannot be generalized to the entire Pakistani population. A detailed and comprehensive analysis of the participants may overcome the limitations of the proposed survey methods. A qualitative research method or mixed research method could prove helpful. Ultimately, the researchers applied only two factors of religiosity, religious commitment and spiritual practice, in this research. Based on previous literature, there are several other aspects of religiosity. Hence, we recommend that these aspects of religiosity may be used as predictor variables in future research. Further research could investigate the relationships between religiosity and marital satisfaction in the context of Pakistani cultural norms.

## Figures and Tables

**Table 1:** Reliability of variables [57].

Sr. No.	Construct	Number of Items	Cronbach’s Alpha
1	Religious commitment (RC)	10	0.797
2	Marital Satisfaction	7	0.750
3	Religious Practice (RP)	4	0.948

**Table 2:** Descriptive Statistics of Variables [57].

Descriptive Statistics of Marital Satisfaction Scale			
	N	Mean	Std. Deviation
I prefer not to show a partner how I feel deep down	508	4.0886	1.36092
I am very comfortable being close to romantic partners	508	2.7972	1.26250
When my partner starts to get close to me, I find myself pulling away	508	4.0728	1.31769
I get uncomfortable when a romantic partner wants to be very close	508	4.1378	1.28130

**Table 3:** Descriptive Statistics of Variables.

	N	Mean	Std. Deviation
Performing Hajj will be my priority the moment I’ve fulfilled all the necessary conditions	508	3.1791	1.43133
I offer/make my prayer always on time	508	3.4843	1.30609
I spend time trying to grow in my understanding of my faith	508	3.3819	1.36302
I believe that Allah helps me	508	3.4764	1.30899

**Table 4:** Descriptive Statistics of Variables.

Descriptive Statistics Religious Commitment Scale			
	N	Mean	Std. Deviation
I make financial contributions to my religious organization	508	3.3465	1.36959
Education level of spouse effects marital satisfaction	508	4.0886	1.36092
I make financial contributions to my religious organization	508	3.3465	1.36959
I make financial contributions to my religious organization	508	3.3465	1.36959
I make financial contributions to my religious organization	508	3.3465	1.36959
I enjoy spending time with others of my religious affiliation	508	2.8839	1.25626
Religious beliefs influence all my dealings in life	508	3.2953	1.44846
I make financial contributions to my religious organization	508	3.3465	1.36959
I prefer not to show a partner how I feel deep down	508	4.0886	1.36092
I turn to my partner for many things, comfort and reassurance	508	4.1437	1.31484

**Table 5:** The correlations between marital satisfaction and religiosity dimensions.

Research Variables	M-Sat.	R-CMT	R-PRCT
Marital satisfaction	1	0.172 ^**^	0.271 ^**^
Religious commitment		1	0.146 ^**^
Religious practice			1

**Note:** * Statistical significance was determined by calculating Pearson’s correlational analysis. **. M-Sat.—marital satisfaction, R-CMT—religious commitment, and R-PRCT—religious practice.

**Table 6:** Regression model for religiosity and marital satisfaction.

Model 1	DV	IV	B	Std. Error	Beta	F	R-square	Adjusted R-Square	Sig
1	MS	RC	0.099	0.025	0.172	15.416	0.030	0.028	0.000
2	MS	RP	−0.016	0.035	−0.020	0.201	0.000	−0.002	0.000
